# The impact of COVID-19 on rare metabolic patients and healthcare providers: results from two MetabERN surveys

**DOI:** 10.1186/s13023-020-01619-x

**Published:** 2020-12-03

**Authors:** C. Lampe, C. Dionisi-Vici, C. M. Bellettato, L. Paneghetti, C. van Lingen, S. Bond, C. Brown, A. Finglas, R. Francisco, S. Sestini, J. M. Heard, M. Scarpa, MetabERN collaboration group

**Affiliations:** 1 Center for Rare Diseases Giessen (ZSEGI), University Hospital Giessen, Giessen, Germany; 2 Division of Metabolism, Bambino Gesù Children’s Hospital, IRCCS, Rome, Italy; 3 MetabERN, Regional Coordinating Center for Rare Diseases, Udine University Hospital, Udine, Italy; 4 Krabbe UK, London, UK; 5 MSD Action Foundation, Dublin, Ireland; 6 Portuguese Association for CDG and Other Rare Metabolic Diseases (APCDG-DMR), Almada, Portugal; 7 Italian Association of Patients With Alkaptonuria (aimAKU), Siena, Italy

**Keywords:** COVID-19, SARS-CoV-2, Coronavirus, Pandemic, Rare diseases, Inherited metabolic diseases, IMD, Survey

## Abstract

The ongoing coronavirus disease 2019 (COVID-19) pandemic has caused disruption in all aspects of daily life, including the management and treatment of rare inherited metabolic disorders (IMDs). To perform a preliminary assessment of the incidence of COVID-19 in IMD patients and the impact of the coronavirus emergency on the rare metabolic community between March and April 2020, the European Reference Network for Hereditary Metabolic Diseases (MetabERN) has performed two surveys: one directed to patients’ organizations (PO) and one directed to healthcare providers (HCPs). The COVID-19 incidence in the population of rare metabolic patients was lower than that of the general European population (72.9 × 100,000 vs. 117 × 100,000). However, patients experienced extensive disruption of care, with the majority of appointments and treatments cancelled, reduced, or postponed. Almost all HCPs (90%) were able to substitute face-to-face visits with telemedicine, about half of patients facing treatment changes switched from hospital to home therapy, and a quarter reported difficulties in getting their medicines. During the first weeks of emergency, when patients and families lacked relevant information, most HCPs contacted their patients to provide them with support and information. Since IMD patients require constant follow-up and treatment adjustments to control their disease and avoid degradation of their condition, the results of our surveys are relevant for national health systems in order to ensure appropriate care for IMD patients. They highlight strong links in an interconnected community of HCPs and PO, who are able to work quickly and effectively together to support and protect fragile persons during crisis. However, additional studies are needed to better appreciate the actual impact of COVID-19 on IMD patients’ health and the mid- and long-term effects of the pandemic on their wellbeing.

## Background

Coronavirus disease 2019 (COVID-19) is a novel infectious disease caused by the severe acute respiratory syndrome coronavirus 2 (SARS-CoV-2), which emerged in China in December 2019. The new coronavirus spread rapidly across the globe, and on 11th March 2020 the World Health Organization (WHO) classified the outbreak as a pandemic [1]. As of 20th October 2020 there have been 40,114,293 confirmed cases of COVID-19 worldwide—of which 8,027,954 in Europe—and 1,114,692 global deaths [2], with different incidence rates and fatalities between countries and regions. Since the pandemic is still ongoing, these numbers are not definitive and, unfortunately, by the time this paper is published the numbers will be higher.

Compared to other coronaviruses such as SARS-1 and MERS, to date SARS-CoV-2 has showed a lower fatality rate (2.3% vs. 9.5% and 34.4%, respectively) [3]. The presence of underlying health conditions, such as diabetes, hypertension and cardiovascular disease, have been correlated with severe COVID-19 and death [4].

The ongoing pandemic of SARS-CoV-2 has suddenly changed all aspects of our daily lives, including how healthcare is organized and delivered. Indeed, the pandemic has severely affected national health systems across Europe: besides the immediate strain put on intensive care units and medical professionals assisting those with COVID-19, most hospitals had to cancel and/or postpone most non-urgent visits, procedures, and therapies, adapting the type and modality of care that they provide to patients affected by all diseases, including those with rare inherited metabolic diseases (IMDs).

IMD patients require constant follow-up and adjustments of treatment and diet, as metabolic destabilization may happen in case of events such as fever or infection. In ordinary situations, IMDs are neglected conditions, with limited resources and attention dedicated to these rare diseases. The COVID-19 pandemic has reduced attention even further. Disruption of the proper follow-up of IMD patients can drastically compromise their clinical condition and aggravate the course of the disease, not only from a medical, but also from a social and psychological point of view, with the possible onset of depression, anxiety, and panic attacks.

Recent surveys from international patients organizations (PO) such as EURORDIS [5] and NORD [6] have revealed the detrimental effects of SARS-CoV-2 on the rare disease community worldwide: from interruption of care and closed hospitals, to medication shortages and significant concern regarding COVID-19 and its impact on the present and future management of rare diseases. Similar results have been observed in national studies conducted in Italy [7] and Ireland [8]. These results show that, in the current emergency situation, surveys are important resources to understand current and future needs of rare disease patients. This information is indeed crucial to organize an appropriate response and to prioritize actions of healthcare providers (HCPs) in the coming months, with the aim of minimizing COVID-19 impact on rare disease patients.

Besides the aforementioned PO, additional surveys have been activated by European Reference Networks (ERNs) to gather more details on the effects of the coronavirus pandemic on specific groups of rare disease patients. Among these, the ERN for Hereditary Metabolic Diseases (MetabERN) has performed two surveys to understand the situation in the rare metabolic community. MetabERN connects centers specialized in rare metabolic diseases at EU level; it represents 77 HCPs from 23 EU Member States and 44 PO, and it is endorsed by the Society for the Inborn Errors of Metabolism (SSIEM). As such, MetabERN is the main reference point and cluster of expertise for the rare metabolic community in Europe.

While information from the patients’ perspective is crucial to understand their unmet medical needs and find appropriate solutions to guarantee their wellbeing, gathering data from HCPs is equally important to have a more comprehensive view of the rare disease community during the ongoing coronavirus emergency. Indeed, HCPs can provide information on the most effective and successful measures that have been implemented to deliver medical care during the pandemic, and can also provide data on the infection rate and the clinical manifestations of SARS-CoV-2 infection in rare disease patients. However, no surveys regarding COVID-19 are currently available from the HCPs point of view.

Here we present the first surveys performed in the field of rare metabolic diseases to analyse the impact of the coronavirus pandemic on the whole metabolic rare disease community: one survey was directed to IMD PO and one was specific for HCPs.

## Methods

The surveys’ design and data collection were performed using the Survey Monkey platform. The survey for HCP (Additional file 1 in the Supplementary Information) consisted of 30 questions and was distributed to all 77 MetabERN centres at the start of the emergency, between 29th March and 17th April 2020. The survey for PO (Additional file 2 in the Supplementary Information) included 32 questions, was active at the beginning of the pandemic, between 26th March and 14th April 2020, and was sent to 45 associations. All data were extracted and analysed using Microsoft Excel. Epidemiological analysis was performed by integrating the survey’s data with the MetabERN database (number of adult and paediatric patients followed by each HCP member of the MetabERN). All participants gave their consent for data collection and publication.

## Results

### HCP survey

#### Incidence of COVID-19 in rare metabolic patients

The results of the HCPs survey were collected from 73 MetabERN units in 21 EU Member States, with a response rate of 95%. Most of these centers (64.4%) assist both adult and paediatric patients, about a quarter (24.7%) provide only paediatric care, and 11% follow adult patients (Table 1).

**Table 1:** Responses collected from the HCPs survey

Category	n (%)
Group of IMD patients followed at the centre	
Adult	8/73 (11)
Paediatric	18/73 (24.7)
Both	47/73 (64.4)
Paediatric patients infected with SARS-CoV-2 (confirmed by testing) in the centre	
Yes	5/73 (6.9)
No	62/73 (84.9)
Do not know	6/73 (8.2)
Total number of paediatric reported cases	13
Symptoms of paediatric patients positive for COVID-19	
At diagnosis	
Asymptomatic	1/13 (8)
Mild	12/13 (92)
Severe	0/13 (0)
Do not know	0/13 (0)
During the infection	
Asymptomatic	1/13 (8)
Mild	12/13 (92)
Severe with need of hospitalisation	0/13 (0)
Required intensive care	0/13 (0)
Adult patients infected with SARS-CoV-2 (confirmed by testing) in the centre	
Yes	7/73 (9.6)
No	54/73 (74)
Do not know	12/73 (16.4)
Total number of adult reported cases	11
Symptoms of adult patients positive for COVID-19	
At diagnosis	
Asymptomatic	0/11 (0)
Mild	10/11 (91)
Severe	1/11 (9)
Do not know	0/11 (0)
During the infection	
Asymptomatic	0/11 (0)
Mild	9/11 (82)
Severe with need of hospitalisation	2/11 (18)
Required intensive care	0/11 (0)
Casualties due to COVID-19 among IMD patients	
Yes	0/73 (0)
No	64/73 (87.7)
Do not know	9/73 (12.3)
Change needed in the management of IMD patients	
Yes	66/73 (90.4)
No	7/73 (9.6)
Change needed in the therapy regime of IMD patients with no COVID-19*	
Yes, the frequency of therapy has been reduced	19/73 (26)
Yes, the therapy has been stopped	3/73 (4.1)
Yes, the frequency of rehabilitation has been reduced	12/73 (16.4)
Yes, rehabilitation has been stopped	15/73 (20.6)
Only for some specific cases	14/73 (19.2)
No	26/73 (35.6)
Changes in therapeutic regimes unified at national or regional level	
Yes	25/45 (55.6)
No	20/45 (44.4)
Proportion of missed outpatient visit for IMD at the centre	
0–25%	5/73 (6.9)
25–50%	3/73 (4.1)
50–75%	20/73 (27.4)
75–100%	40/73 (54.8)
Not applicable	5/73 (6.9)
Outpatient face-to-face visits replaced by video conference/telephone interaction	
Yes	66/73 (90.4)
No	4/73 (5.5)
Not applicable	3/73 (4.1)
Patients stopped treatment by their own decision	
Yes	10/73 (13.7)
No	57/73 (78.1)
Do not know	6/73 (8.2)
Disease categories expected to be at major risk in relation to COVID-19*	
AOA	42/73 (57.5)
PM-MD	41/73 (56.2)
C-FAO	33/73 (45.2)
LSD	38/73 (52)
PD	14/73 (19.2)
CDG	18/73 (24.7)
NOMS	9/73 (12.3)
Awareness of good-quality informative material about COVID-19 and IMD	
Yes	26/73 (35.6)
No	33/73 (45.2)
Do not know	14/73 (19.2)
Centre produced informative material about COVID-19 and IMD	
Yes	27/73 (37)
No	42/73 (57.5)
Do not know	4/73 (5.5)
Centre offering special informative/psychological support to IMD patients during the pandemic	
Yes	43/73 (58.9)
No	24/73 (32.9)
Do not know	6/73 (8.2)
PO helping HCP in providing special support during the pandemic	
Yes	42/73 (57.5)
No	21/73 (28.8)
Do not know	10/73 (13.7)
Active COVID-19 helpline for IMD patients in the centre	
Yes	38/73 (52.1)
No	32/73 (43.8)
Do not know	3/73 (4.1)
Patients prone to metabolic crises have the same open access to hospital as before the COVID-19 outbreak	
Yes	49/73 (67.1)
No	18/73 (24.7)
Do not know	6/73 (8.2)

^*^More than one answer possible

AOA, amino and organic acids-related disorders; PM-MD, disorder of pyruvate metabolism, Krebs cycle defects, mitochondrial oxidative phosphorylation disorders, disorders of thiamine transport and metabolism; C-FAO, carbohydrate, fatty acid oxidation and ketone bodies disorders; LSD, lysosomal storage disorders; PD, peroxisomal disorders; CDG, congenital disorders of glycosylation and disorders of intracellular trafficking; NOMS, disorders of neuromodulators and other small molecules

As of 17th April 2020, only 13 paediatric cases of COVID-19 were registered in just 7% of centres: at the time of diagnosis and also during the course of infection, 12 patients showed mild symptoms of the disease, while one patient was asymptomatic (Table 1). These paediatric cases were located in Italy (1/13), the Netherlands (1/13), and mostly in France (11/13).

In mid-April 2020, only about 10% of centers had COVID-19 cases among adult patients, for a total of 11 cases: at diagnosis, the symptoms were mild in 10 patients and severe in one case; during the infection, nine patients showed mild symptoms, and two expressed severe symptoms and were hospitalised (Table 1). Similarly to paediatric cases, the majority of adult IMD patients with COVID-19 were reported in France (6/11), followed by the UK (2/11), Belgium (2/11), and Sweden (1/11).

No COVID-19-related deaths were reported among IMD patients (Table 1). The survey indicates an incidence of COVID-19 infection of 24/32,936 patients (72.9/100,000 equivalent for paediatric and adults, 95% CI: 46.5–106.8/100,000) in the European IMD community.

**Fig. 1:**
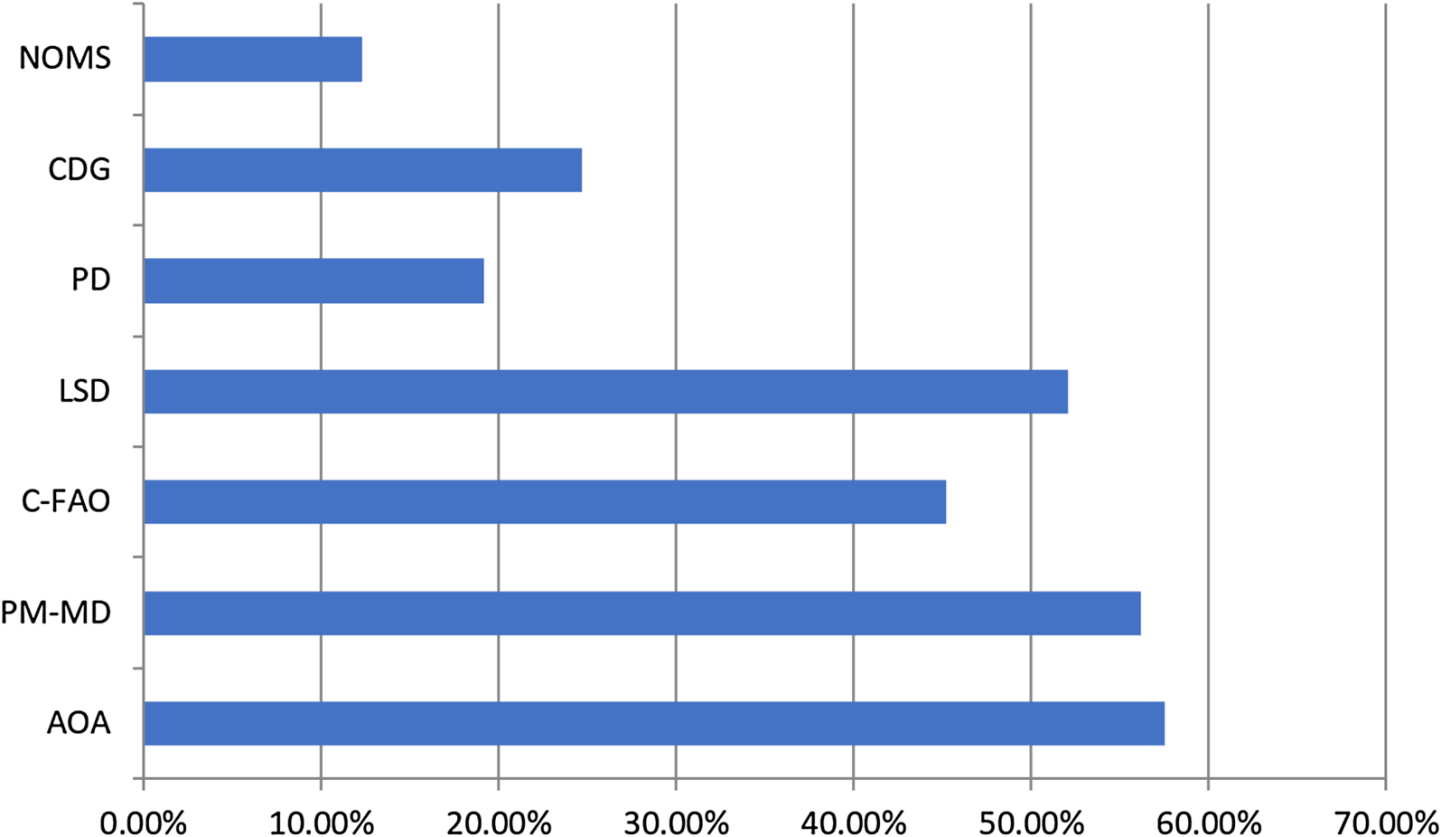
Disease categories considered at major risk for severe COVID-19 (% of centers considering the disease at major risk)

At the beginning of the pandemic, HCPs expressed some concerns regarding the risks of severe forms of COVID-19 in IMD patients; indeed, the following IMDs are considered a major risk factor for the coronavirus disease by MetabERN experts (Table 1 and Fig. 1): amino and organic acids-related disorders (AOA) (at high risk according to 57.5% of centers); disorders of pyruvate metabolism, Krebs cycle defects, mitochondrial oxidative phosphorylation disorders, disorders of thiamine transport and metabolism (PM-MD) (56.2%); lysosomal storage disorders (LSD) (52%); and carbohydrate, fatty acid oxidation and ketone bodies disorders (C-FAO) (45.2%).

#### Management of rare metabolic patients during the emergency

**Fig. 2:**
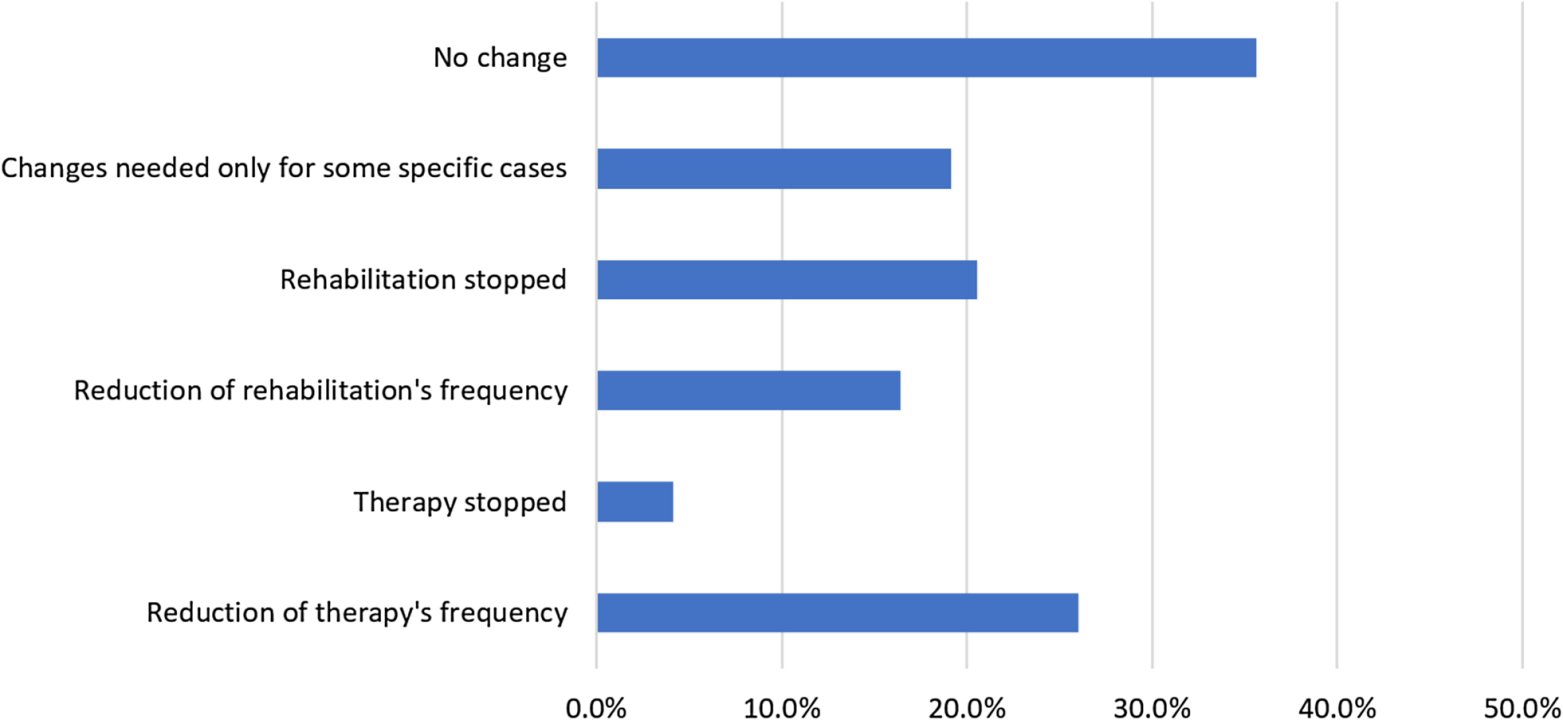
Changes in IMD patients’ therapy regimen at the beginning of the COVID-19 emergency (% of centers; more than one answer possible)

Ninety percent of MetabERN centers had to change the management of their patients due to the ongoing pandemic. In particular, changes in therapy regimen were reported by 64.4% of centers: the frequency of therapy and of rehabilitation had to be reduced in 26% and 16.4% of centers, respectively, and in 21% of centers rehabilitation was stopped (Table 1 and Fig. 2).. Where changes in the therapeutic regimes were put into place, these were unified at a regional or national level in over half of the centers (55.6%) (Table 1). Most IMD patients (78.1%) did not stop treatment on their own, however 14% did (Table 1).

**Fig. 3:**
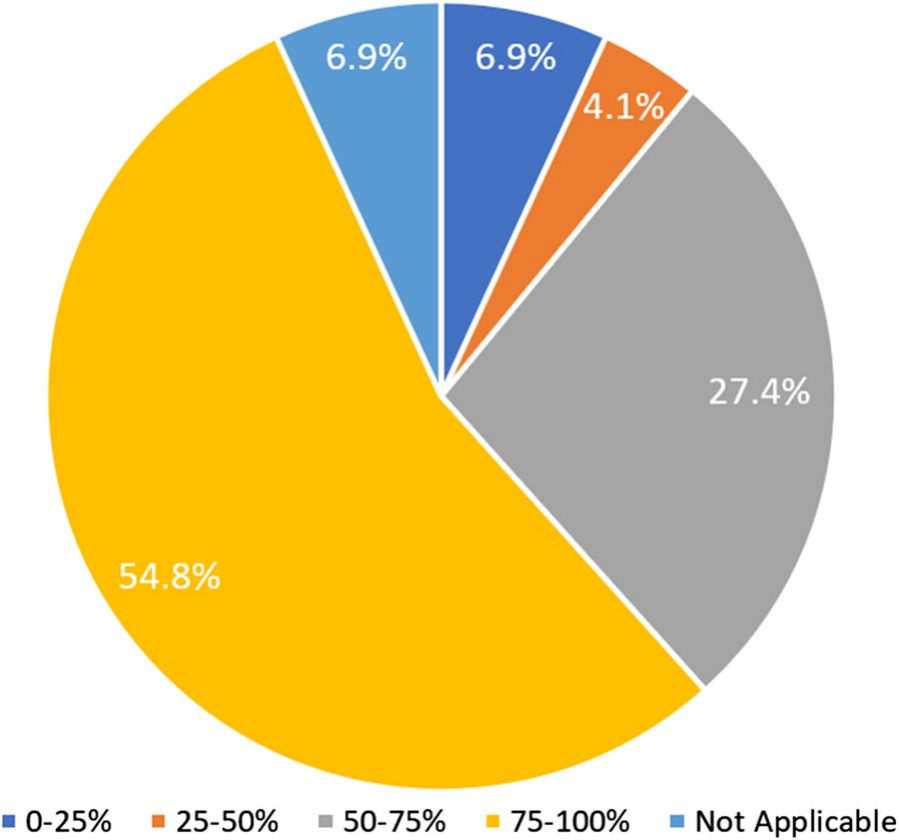
Proportion of missed outpatient visits at the centre

The proportion of missed outpatient visits for IMDs was relevant, with 82% of centers reporting between 50 and 100% of missed visits (Table 1 and Fig. 3). However, almost all visits (90.4%) were replaced by video or phone consultations (Table 1).

Despite the present emergency, the majority of HCPs (67.1%) believe that patients with conditions prone to metabolic crises had the same access to hospitals as before the COVID-19 outbreak (Table 1).

#### Information about COVID-19 in the rare metabolic community

At the beginning of the emergency, good-quality, IMD-specific informative material about COVID-19 from local, national, or institutional sources was missing: many MetabERN members (45.2%) were not aware of this kind of information being available, and most centers (57.5%) had not yet produced dedicated material to inform their patients (Table 1). However, in the ongoing pandemic the majority of centers (58.9%) offered special informative and psychological support directly to their IMD patients, mostly with the help of PO (57.5%) (Table 1). In addition, over half of the MetabERN centers (52%) activated a COVID-19 helpline for their metabolic patients (Table 1).

### PO survey

#### Incidence and management of COVID-19 in rare metabolic patients

Data relevant to the patients’ perspective were collected from 39 metabolic PO from 18 EU countries, with an 87% response rate. Similarly to HCPs, the majority of PO (87.2%) reported no cases of COVID-19 among metabolic patients (Table 2). The PO that were aware of patients with COVID-19 in their IMD community were located in Denmark (1/5), Austria (1/5), the Netherlands (1/5), Belgium (1/5) and Italy (1/5) (no specific number of patients affected available).

**Table 2:** Responses collected from the PO survey

Category	n (%)
PO contacted by/know of IMD patient with COVID-19	
Yes	5/39 (12.8)
No	34/39 (87.2)
PO and/or its members in close contact with the specialised centre/HCP about the COVID-19 crisis	
Yes	26/39 (66.7)
No	7/39 (18)
Do not know	6/39 (15.3)
PO informed by specialist centre about COVID-19 and its risk for IMD patients	
Yes	27/37 (73)
No	10/37 (27)
Centers following IMD patients in the country that are in contact with the PO	
All of them	10/36 (27.8)
Most of them (≥ 50%)	8/36 (22.2)
A few (< 50%)	10/36 (27.8)
None	8/36 (22.2)
Centers have put into place measures or alternative pathways to prevent COVID-19 in IMD patients	
Yes, all of them	10/37 (27)
Yes, some of them	8/37 (21.6)
Almost none	2/37 (5.4)
None	4/37 (10.8)
Do not know	13/37 (35.1)
PO’s members experiencing changes	
In follow up visits at the specialised centre	
Yes	32/37 (86.5)
No	1/37 (2.7)
Do not know	4/37 (10.8)
In follow up visits for clinical trials at the specialised centre	
Yes	14/30 (46.7)
No	3/30 (10)
Do not know	13/30 (43.3)
Type of changes*	
Cancellations of outpatient stays	17/31 (54.8)
Cancellations of inpatient stays	10/31 (32.3)
Postponing of appointments	28/31 (90.3)
Changes in IMD treatment due to COVID-19 pandemic	
Yes	20/39 (51.3)
No	19/39 (48.7)
Type of changes*	
Discontinuation	13/20 (65)
Prolonged time between treatments	7/20 (35)
Switch to home therapy	10/20 (50)
Problems in getting the medication	5/20 (25)
Therapies (physio, speech, or enzyme replacement therapy, etc.) continued	
Yes	10/38 (26.3)
No	16/38 (42.1)
Do not know	12/38 (31.6)
Psychological support available for patients scared about COVID-19	
Yes	28/39 (71.8)
No	11/39 (28.2)
Specialist/entity providing psychological support*	
Physician	8/32 (25)
Psychologist	11/32 (34.4)
Social worker	7/32 (21.9)
PO	21/32 (65.6)
Other	13/32 (40.6)
Method/technology used to provide psychological support*	
Phone	25/31 (80.7)
Mail/Email	20/31 (64.5)
Video	15/31 (48.4)
Other	9/31 (29)

^*^More than one answer possible

Most organizations (73%) (and/or their members) have been informed by specialized centres about COVID-19 and its risks for IMD patients, and the majority (66.7%) also say that their patient members are in close contact with their specialized centre/HCP about the COVID-19 crisis (Table 2).

Most associations (78%) have been contacted by some or all hospital centers in their country following their IMD members, and many organizations (48.6%) know of HCPs that have introduced special measures or alternative care pathways to prevent SARS-CoV-2 infection in metabolic patients (Table 2): video or phone consultations, transfer to the hospital with a taxi or emergency transport, and home therapy.

##### Variations in visits and treatments

**Fig. 4:**
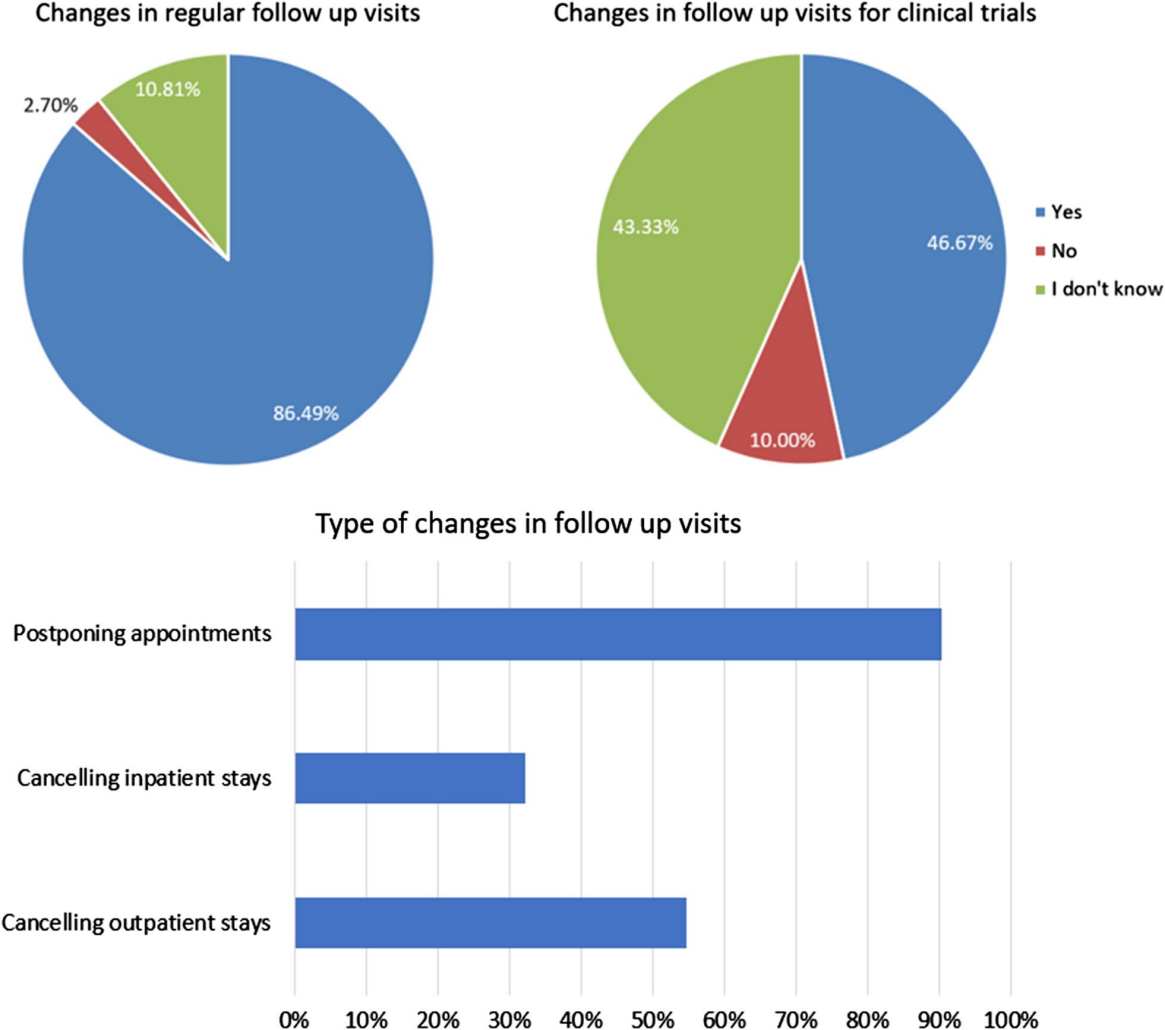
Proportion (top) and type of changes (bottom) in regular and clinical trials follow-up visits at the specialised centres

Changes were reported in 86.5% of follow-up visits at specialized centers and in 47% of follow-up visits for clinical trials, with respectively 55% and 32% of cancelled outpatients and inpatient stays, and 90% of postponed appointments (Table 2 and Fig. 4).

**Fig. 5:**
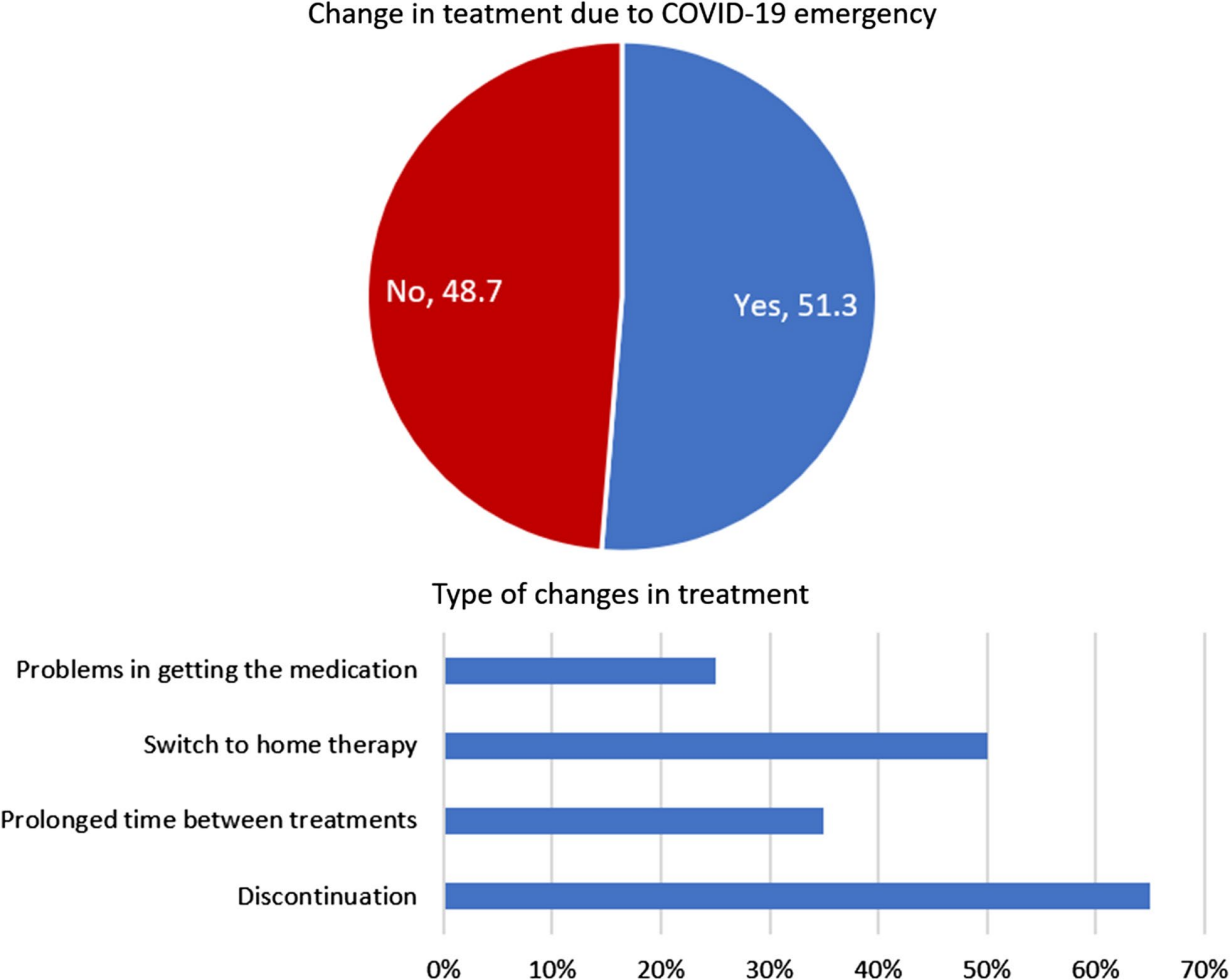
Proportion (top) and type of changes (bottom) in IMD treatment due to the COVID-19 emergency

Over half of PO (51.3%) reported changes also in IMD treatments due to COVID-19, in particular: discontinuation (65%), change to home treatment (50%), prolonged time between treatments (35%), and problems in getting the medication (25%) (Table 2 and Fig. 5). Only 26% of organizations reported no interruption of therapy (e.g. physiotherapy, speech therapy, enzyme replacement therapy, etc.), while 42% said these were stopped (Table 2).

##### Support and concerns about COVID-19 in rare metabolic patients

Seventy-two percent of PO confirmed that psychological support was provided to patients worried about the COVID-19 outbreak, mostly through the PO themselves (65.6%) via phone, video, or email (Table 2).

**Fig. 6:**
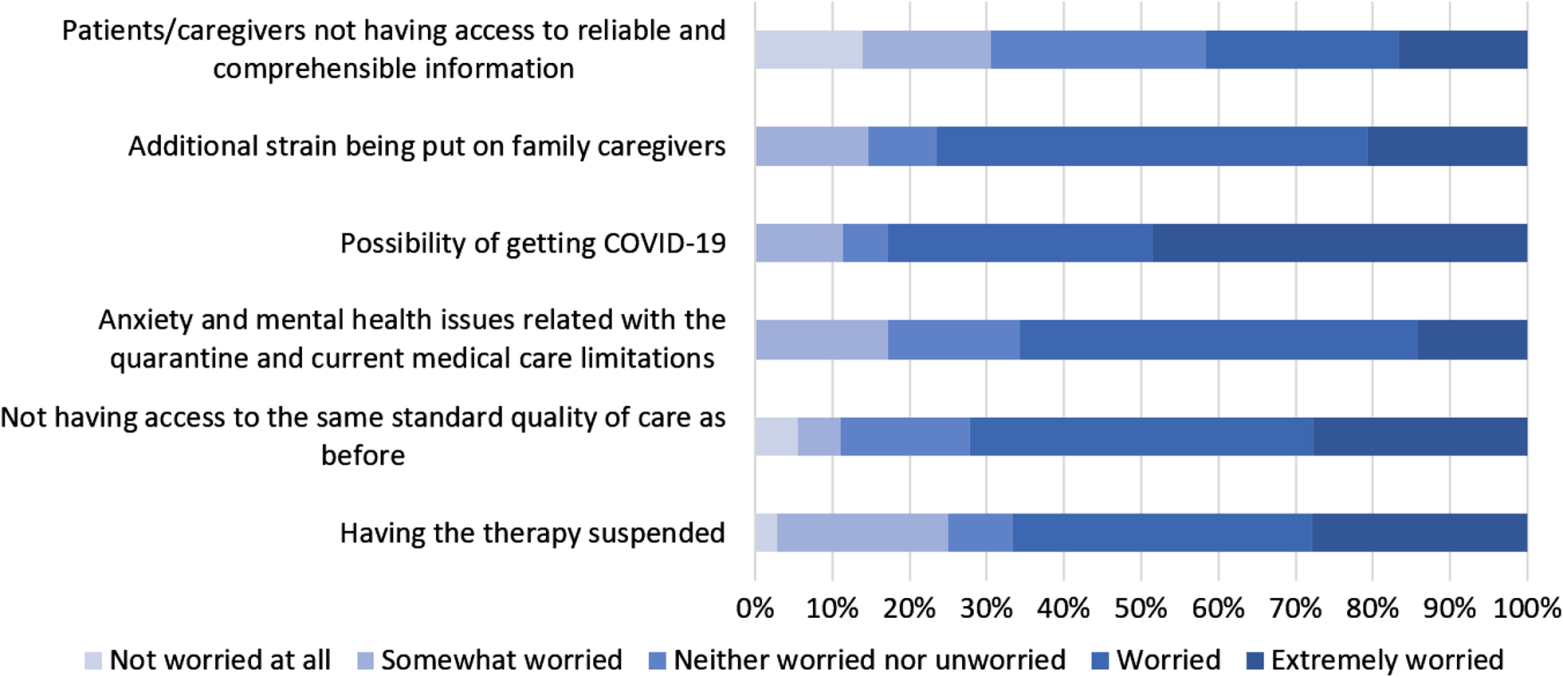
Type and level of concern of the IMD patient community represented by the PO

According to PO, besides the possibility of being infected with COVID-19, most patients were also worried about not having access to therapy or to the same quality of care as before. In addition they were concerned about additional pressure put on family caregivers, anxiety and mental health issues related to quarantine and care limitations (Table 3 and Fig. 6).

**Table 3:** Description and levels of concern of the IMD patient community

Description of concern	Level of concern				
	Not worried at all	Somewhat worried	Neither worried nor unworried	Worried	Extremely worried
Having the therapy suspended	1/3 (2.8)	8/36 (22.2)	3/36 (8.3)	14/36 (38.9)	10/36 (27.8)
Not having access to the same standard quality of care as before	2/36 (5.6)	2/36 (5.6)	6/36 (16.7)	16/36 (44.4)	10/36 (27.8)
Anxiety and mental health issues related with the quarantine and current medical care limitations	0/35 (0)	6/35 (17.1)	6/35 (17.1)	18/35 (51.4)	5/35 (14.3)
Possibility of getting COVID-19	0/35 (0)	4/35 (11.4)	2/35 (5.7)	12/35 (34.3)	17/355 (48.6)
Additional strain being put on family caregivers	0/34 (0)	5/34 (14.7)	3/34 (8.8)	19/34 (55.9)	7/34 (20.6)
Patients/caregivers not having access to reliable and comprehensible information	5/36 (13.9)	6/36 (16.7)	10/36 (27.8)	9/36 (25)	6/36 (16.7)

## Discussion

The coronavirus pandemic has taken healthcare system all over the world by surprise: totally unprepared, HCPs had to rapidly change the way they organize and deliver medical care, not only to assist COVID-19 cases, but for all patients. As such, the pandemic is having a significant impact on patients affected by chronic and multiorgan disorders such as IMDs. In the general population, the most severe and fatal cases of COVID-19 are observed in patients 60+ years of age and in those with underlying health conditions (diabetes, hypertension, cardiac disorders, chronic lung disease), while children are less frequently infected and present milder symptoms than adults [4]. In the rare metabolic community, where all patients, including pediatric ones, experience multiorgan impairments caused by their chronic condition, the risk of severe forms of COVID-19 may be high. Indeed, at the early stage of the pandemic, MetabERN experts were concerned regarding the potential effect of the infection on their patients, as those with an IMD have a genetic condition that predisposes them to metabolic destabilization and gradual worsening of the clinical course, with progressive neurological deterioration and multi-organ dysfunction; for these reasons, constant controls, diet corrections, and therapy are critical for the wellbeing and, in some cases, survival of IMD patients. Therefore, recommendations to decrease the infection rate were expressed by the network.

In the present situation, rare metabolic patients need to be protected not only from the risk of coronavirus infection, but also from the possible degeneration of their disease. To this end, a network of HCPs in close contact with IMD patients and their caregivers is essential to provide patients with the necessary support and care. Indeed, our two surveys revealed a close interaction of HCPs with their patients and the PO during this time of crisis. At the same time, the HCPs survey showed that at the beginning of the coronavirus emergency (March–April 2020) the incidence of COVID-19 among IMD patients was lower than that of the general European population (EU/EEA and UK) reported from 1st January to 7th April 2020 (72.9/100,000 vs. 117/100,000) [9]. Both aspects suggest the existence of a strong IMD community, supported by a solid network of HCPs and PO that were able to act quickly and effectively to protect metabolic patients from SARS-CoV-2 infection—at least to a certain extent. In an unprecedent situation with a lot of unknowns, during which specific information from health institutions and scientific literature about COVID-19 in metabolic diseases was not available or limited, HCPs contacted the patients directly to provide the necessary support and information. As the situation rapidly evolved, informative material on coronavirus and IMDs became available from local and international institutions and organizations; MetabERN collected the links to these resources on its website and also published recommendations, Q&A and infographics about COVID-19 in rare metabolic patients [10]. In general, IMD patients receive detailed information from specialists to avoid any metabolic decompensation due to other diseases or infections. For COVID-19, it was not necessary to provide additional indications besides the general information given at the local or national level. Importantly, to help the entire metabolic community, MetabERN has developed some recommendations to support all IMD patients and caregivers during the COVID-19 emergency. These recommendations stress the importance of sustaining efforts to prevent, diagnose and treat IMDs, assuring the continuation of the quality of care provided to patients and recommending, whenever possible, to direct patients to home therapy and to invite them to go to the hospital for a visit only if really needed. In particular, the use of videoconferences as a replacement for many of the face-to-face meetings is encouraged.

At the very beginning HCPs did not know what kind of disease COVID-19 was, so the information was based on avoiding decompensation. Now that HCPs know what disease it is, they give more precise recommendations on how to manage fever to avoid metabolic decompensation.

According to the data collected through our HCPs survey, COVID-19 cases in IMD patients were registered in six EU countries: Belgium, Italy, the Netherlands, Sweden, the UK, and especially France, where most of the cases were reported (17 out of 24). This may suggest that the IMD community in France was more heavily hit by the pandemic than the rest of the EU; however, these results may be linked to a better identification of COVID-19 cases in France compared to other countries thanks to a more rigorous reporting to HCPs by patients, a better reporting of cases to MetabERN by French specialists, or an actual higher number of cases. Unfortunately, our data are too small to reach statistical significance, so they cannot be used to make comparisons or draw any conclusions on the national incidence of COVID-19 among IMD patients. In addition, our data may be incomplete and reflect only a fraction of the national situation reported by MetabERN centres; plus, in some European countries testing for COVID-19 might have been limited or unavailable at the time of our survey.

HCPs data on COVID-19 cases differ from that collected from the PO, which reported COVID-19 patients within the IMD community in five countries: Denmark, Austria, the Netherlands, Belgium and Italy—notably, not France. This result suggests that, while communication between HCPs and PO on the patients’ management in general and in emergency situations is rapid and up-to-date, communication on specific clinical cases is lacking. Also, it is difficult to define the interaction between HCPs and PO, and between PO and patients: some PO interact often with HCPs and their patients, others do not; similarly, not all patients are in regular contact with their association.

As a mean to protect IMD patients from COVID-19, visits were mostly cancelled or postponed: more than half of HCPs reported 75–100% of missed outpatient visits, confirming data gathered from the PO, which reported changes in 87% of follow-up visits (and in 47% of clinical trials visits), with 90% of postponed appointments and 55% of cancelled outpatients stays. These results are in line with other surveys performed on the broader rare disease community by EURORDIS, NORD and Rare Disease Ireland. According to these studies, 80% of patients had their rehabilitation postponed or cancelled [5], and 50–70% had their appointments with the general practitioner or specialist cancelled [5, 6, 8]. As SARS-CoV-2 reached Europe and governments and healthcare systems struggled to contain the spread of the virus, cancelling or postponing face-to-face visits was the most obvious, immediate and critical action to put into place to limit infections and protect all patients, including those with IMDs.

Similarly, most treatments were reduced or suspended: a quarter of HCPs reduced the frequency of therapy, stopping it in only 4% of cases, while rehabilitation was reduced or stopped in 16% and 21% of cases, respectively. Indeed, according to the PO, over half of IMD patients experienced a change in treatment: 65% faced discontinuation; 50% switched to home therapy; 35% prolonged the time between treatments; and 25% had problems in getting the medication. The results on the discontinuation of care are in line with recent data about the effects of the pandemic in Italy, where 52% of rare disease patients suspended hospital therapy [7] and 49% of LSD patients receiving enzyme replacement therapy in hospital experienced disruption [11]. Considering the huge impact of COVID-19 on hospitals’ organization and workload, it is not surprising that in many cases treatments for rare disease patients had to be halted or rearranged to limit the infection and to divert resources towards COVID-19 patients. However, our results are somehow different from those of the EURORDIS survey: while this reported that 60% of rare disease patients had no access to therapies such as infusions, chemotherapy, and hormonal treatment at home or at the hospital [5], we found that about half of IMD patients were able to switch to home therapy. This difference may be due to the fact that home treatment is an option for IMDs, but not for other rare diseases. Also, national healthcare systems in EU countries were re-organised differently during the emergency, and this may have impacted the availability of personnel, medical equipment and drugs for rare diseases.

Our data also show that about a quarter of patients are encountering difficulties in accessing medicines. This result is similar to what was reported in Ireland [8] and Italy [7] for rare diseases in general, but it differs from the situation of LSD therapies in Italy, where there were no problems in medicine supply, even in the most affected regions [11]. These differences are most likely a reflection of the different national healthcare settings across Europe and of the different drugs used by rare diseases patients. Still, the situation is worrisome and calls for an immediate and coordinate action by national health systems.

Importantly, our surveys show that in most cases (78%) rare metabolic patients did not stop treatment by their own decision: a higher percentage than that reported in the Italian survey on rare disease patients (44%) [7]. This aspect is a further evidence of the strong collaboration that exists between HCPs and IMD patients.

The immediate consequences of visits and treatments disruption for the patients were mitigated by the widespread use of telemedicine among HCPs, as 90% of centres were able to replace face-to-face visits with video or phone calls during the emergency. This percentage is much higher than that observed in the general rare disease community in Europe and in the United States, where 30–60% of patients used telemedicine or other forms of remote consultation [5, 6, 8]. With telemedicine becoming more and more relevant for the future of healthcare, reducing physical and geographical barriers, this result demonstrates that the IMD community is ready to adopt current and novel digital tools to ensure constant and adequate care to all metabolic patients.

The main fears of IMD patients include getting COVID-19, not having access to the same quality of care as before the pandemic, and having the therapy suspended. In normal circumstances, rare metabolic patients face constant challenges to maintain their wellbeing and avoid a degeneration of their condition, so these results are not surprising given the significant changes imposed by the pandemic on all aspects of daily life. Indeed, other surveys found that the majority of rare disease patients are worried about the impact of COVID-19 on their disease—being it direct infection and/or indirect interference with the management and treatment of the disease [5, 6, 8]. The concerns of IMD patients are somewhat in line with those expressed by cancer and diabetic patients in Europe: recent surveys have showed that people with cancer in the Netherlands and the UK are mostly concerned about the impact of the pandemic on their treatment or follow-up [12] and are worried about catching COVID-19 and becoming seriously ill from this disease [13]; similarly, the main concern of patients with type 1 diabetes in Denmark is to be overly affected due to diabetes if infected with SARS-CoV-2 [14].

The model of care established within the rare metabolic community is based on a close collaboration between HCPs, patients and local PO; during the unprecedent crisis caused by SARS-CoV-2, the results of our surveys suggest that this model was successful in protecting IMD patients from the infection. More time is needed to determine the incidence of COVID-19 in rare metabolic patients and to fully assess the mid- and long-term impact of the pandemic on their wellbeing. As a first step in addressing this issue, we have already developed a second survey for HCPs to better understand the number of COVID-19 cases in IMD patients, and the clinical manifestations and consequences of the infection in these patients across Europe. As the pandemic progresses, various measures can be put into place to mitigate its impact on IMD patients and alleviate the shortcomings identified in our surveys: each hospital should setup appropriate and well-identified COVID-19-free paths and areas to allow a safe use of healthcare services by patients not infected by SARS-CoV-2; home therapy should be expanded as much as possible to ensure treatment to all patients; telemedicine tools should be implemented widely to provide clinical and also psychological support to patients and their caregivers; and additional daily assistance (through nurses or social assistants) should be offered to those with neurological and/or highly debilitating IMDs.

Our study has several limitations: first, as mentioned above, the data collected is too small to make significant statistical analyses focused on the single country or region; therefore, while we were able to identify some trends at a European level, we cannot make any reliable considerations at a national level. Furthermore, the data were generated inside the MetabERN, so we cannot expand our conclusions outside our network of HCPs and patients. Secondly, we were able to collect only qualitative data on the risk of severe COVID-19 in IMD patients: HCPs gave their opinion on whether having an IMD increases this risk, but no quantitative data is currently available to support these opinions. To gather the necessary quantitative data we have already distributed among MetabERN members a second survey on COVID-19, which focuses on the epidemiology and the clinical consequences of SARS-CoV-2 infection in rare metabolic patients. Thirdly, while the PO survey was extremely useful to understand the impact of the coronavirus pandemic from the patients’ perspective, the data collected is limited to the amount of information available to the PO, which may be incomplete and not up to date compared to what HCPs know.

The continuing disruption of the proper follow-up of IMD patients might compromise their clinical condition and aggravate the course of the metabolic disease, from a medical, psychological, and also social point of view. Therefore, HCPs and PO need to keep working together to monitor the health status of their patients and provide the necessary care using the available telemedicine tools; at the same time, long-term adjustments must be put into place by national health systems to restore continuous therapies and treatment for IMD patients.

## Conclusion

We provide the first and most comprehensive evidence of the impact of COVID-19 on the rare metabolic community. During the initial phase of the coronavirus pandemic, the highly medically supervised population of IMD patients was relatively protected from the infection. This was made possible also by the widespread use of telemedicine in the metabolic community. Indeed, digital tools have, to some extent, the capacity to ensure the continuity of care, not only in critical situations like a pandemic, but also for standard healthcare. More time is needed to determine the actual consequences of the COVID-19 emergency on IMD patients.

## Supplementary information

**Additional file 1.** Original survey sent to HCPs. List of questions and possible answers included in the HCPs survey.

**Additional file 2.** Original survey sent to PO. List of questions and possible answers included in the PO survey.

## Data Availability

The datasets used and/or analysed during the current study are available from the corresponding author on reasonable request.

## Abbreviations

WHO: World Health Organization
IMD: Inherited metabolic diseases
PO: Patients organizations
HCP: Healthcare provider
ERN: European Reference Network
MetabERN: European Reference Network for Hereditary Metabolic Diseases
