# Supplementary material for: The impact of COVID-19 on rare metabolic patients and healthcare providers: results from two MetabERN surveys
Source: Orphanet J Rare Dis. 2020 Dec 3;15:341. doi: 10.1186/s13023-020-01619-x (PMC7711270; doi:10.1186/s13023-020-01619-x)
Supplement: Supplementary file 1 — Additional file 1. Original survey sent to HCPs. List of questions and possible answers included in the HCPs survey. [file 13023_2020_1619_MOESM1_ESM.pdf]

Introduction

This survey contains questions inquiring about the impact of the Coronavirus (Covid-19) infection on the management and care of patients affected by Inherited Metabolic Diseases (IMDs) in your Health Care Provider (HCP).

The purpose of this survey is to:

a) Have a first overview of how the different centers are maintaining healthcare services during the ongoing COVID-19 pandemic;
b) Understand whether IMDs patients might be at major risk of complications;
c) To start setting up plans and protocols as well as;
d) To share proper informative material for metabolic patients on the MetabERN website.

This is a first survey; we are planning to send a follow-up one in the next weeks that will focus on other, more specific aspects related to more clinical aspects of the COVID-19 infection in patients with IMDs.

The final goal is to write a paper on the prevalences of infection / disease expression / complications/management in patients with IMDs compared to the general population.

The estimated time to complete the whole survey is 6 minutes.

* 1. Please fill in your name, country, and mail address.

Name ______

Country ______

Email Address ______

* 2. Please select the name of your hospital /institute from the drop-down menu:

______

* 3. Please select your group of patients:

- ○ Adult
- ○ Paediatric
- ○ Both

* 4. Have you had paediatric IMD patients infected by Coronavirus (Covid-19) (confirmed by testing) in your center?

- ○ Yes
- ○ No
- ○ I don't know

5. If yes how many? (please indicate the number)

0 — 100 ______

* 6. Your paediatric IMD patients positive to Covid-19 were:

- ◯ Asymptomatic when diagnosed
- ◯ Had mild disease when diagnosed
- ◯ Had severe disease when diagnosed
- ◯ I don't know
- ◯ Not Applicable

* 7. Your paediatric IMD patients positive to Covid-19:

- ◯ Remained asymptomatic
- ◯ Expressed mild symptoms
- ◯ Expressed severe symptoms and were hospitalized
- ◯ Received intensive care
- ◯ Not Applicable

* 8. Have you had adult IMD patients infected by Covid-19 (confirmed by testing) in your center?

- ◯ Yes
- ◯ No
- ◯ I don't know

9. If yes how many?

0 100

* 10. Your adult IMD patients positive to Covid-19 were

- ◯ Asymptomatic when diagnosed
- ◯ Had mild symptoms when diagnosed
- ◯ Had severe symptoms when diagnosed
- ◯ I don't know
- ◯ Not Applicable

* 11. Your adult patients positive to Covid-19

- ◯ Remained asymptomatic
- ◯ Expressed mild symptoms
- ◯ Expressed severe symptoms and were hospitalized
- ◯ Received intensive care
- ◯ Not Applicable

12. Do you think that being an IMD-patient may increase the risk of being infected by Covid-19?

- ◯ Yes
- ◯ No
- ◯ I don't know

* 13. Have you had casualties among your IMD patients due to COVID-19 infection?

- ◯ Yes
- ◯ No
- ◯ I don't know

* 14. Did the management of your patients require any change? (DH or Ambulatory cancelation, cancelation of programmed visits)

- ◯ Yes
- ◯ No

* 15. Did you have to change the therapy regime for IMD patients unaffected by Covid-19? (frequency of therapy/ Rehabilitation, cancellation of therapy/Rehabilitation)

- [ ] Yes, the frequency of therapy has been reduced
- [ ] Yes, the therapy has been stopped
- [ ] Yes, the frequency of rehabilitation has been reduced
- [ ] Yes, rehabilitation has been stopped
- [ ] Only for some specific cases (please specify below)
- [ ] No
- [ ] please specify

* 16. If you replied yes to the questions above, have changes in the therapeutic regimes been unified at a regional or national level?

- ○ Yes
- ○ No
- ○ Not Applicable

* 17. What is the proportion of missed outpatient visits for IMD at your Center?

- ○ 0-25%
- ○ 25-50%
- ○ 50-75%
- ○ 75-100%
- ○ Not Applicable

* 18. Regarding the missed outpatient visits: Do you replace face-to-face consultations with video conference/telephone with patients?

- ○ Yes
- ○ No
- ○ Not Applicable

* 19. Are you aware of patients at your center that had to change their therapy regime? (Frequency of therapy, cancellation of therapy)

- ○ Yes
- ○ The patients opted out
- ○ The Covi-19 regulations in society prevented it
- ○ No
- ○ I don't know

* 20. Did patients stop treatment on their own?

- ○ Yes
- ○ No
- ○ I don't know

* 21. Please choose the disease category/ies you expect at major risk in relation to Covid-19 infection in itself (given that treatment, eg ERT is given as usual):

- [ ] 1. Amino and organic acids-related disorders (AOA)
- [ ] 2. Disorder of pyruvate metabolism, Krebs cycle defects, mitochondrial oxidative phosphorylation disorders, disorders of thiamine transport and metabolism (PM-MD)
- [ ] 3. Carbohydrate, fatty acid oxidation and ketone bodies disorders (C-FAO)
- [ ] 4. Lysosomal storage disorders (LSD)
- [ ] 5. Peroxisomal disorders (PD)
- [ ] 6. Congenital disorders of glycosylation and disorders of intracellular trafficking (CDG)
- [ ] 7. Disorders of Neuromodulators and Other Small Molecules (NOMS)

* 22. Are you aware of informative material about Covid-19 and metabolic diseases that has been made available publicly and that you believe is good quality information?

- ○ Yes
- ○ No
- ○ I don't know

* 23. Did your Center prepare informative material about COVID-19 and metabolic diseases ?

- ○ Yes
- ○ No
- ○ I don't know

* 24. If yes, would you share informative material about Covid-19 with MetabERN for publication on the web? (If yes, please send the informative material to cinzia.bellettato@metab.ern-net.eu)

- ○ Yes
- ○ No

* 25. Is your center offering special informative/physiological support to your IMDs patients in case of Covid-19 pandemic?

- ○ Yes
- ○ No
- ○ I don't know

* 26. Are Patient Associations helping your center with this?

- ○ Yes
- ○ No
- ○ I don't know

27. Do you have an active COVID-19 helpline for IMDs patients in your center?

- ○ Yes
- ○ No
- ○ I don't know

* 28. Do you have an active Covid-19 helpline specifically for IMD patients in your center?

- ○ Yes
- ○ No
- ○ I don't know

* 29. If yes and if you agree please share contact with us (send it via e-mail to cinzia.bellettato@metab.ern-net.eu)

- Yes
- No

* 30. Do you think patients with conditions prone to metabolic crises have the same open access to hospital as before the Covid-19 outbreak?

- Yes
- No
- I don't know
