# Supplementary material for: The impact of COVID-19 on rare metabolic patients and healthcare providers: results from two MetabERN surveys
Source: Orphanet J Rare Dis. 2020 Dec 3;15:341. doi: 10.1186/s13023-020-01619-x (PMC7711270; doi:10.1186/s13023-020-01619-x)
Supplement: Supplementary file 2 — Additional file 2. Original survey sent to PO. List of questions and possible answers included in the PO survey. [file 13023_2020_1619_MOESM2_ESM.pdf]

Covid-19: From the patients' perspective

Dear Patient Representative,

Due to the COVID-19 pandemic, we are all experiencing unexpectedly a very uncertain, scary, shocking and unpredictable situation all over the world. This is true not only in a medical and economical but also in a psychological sense.

In particular patients with rare metabolic diseases and chronic diseases may have a higher risk of being sick due to COVID-19. At least they may have some unexpected additional complications in getting information, visits, treatment, therapies, rehabilitation etc.

MetabERN wants to support and help!

On a European level, we are stronger and more powerful- rare diseases are already the orphans- we now need to merge our knowledge, experience and our expertise to maintain the standard of care for rare disease patients as much as possible and provide the necessary support especially in such challenging times.

To understand the situation of metabolic patients in different countries and different metabolic disease groups, and to provide useful and concrete information and recommendations that facilitate the already difficult life with a rare disease, we kindly ask you to fill in the attached questionnaire on behalf of your organisation.

Time commitment: 10 min

Deadline: 10 April 2020

Please help us so that we can help you!

We wish you and your families good health and the power to get through these unprecedented times.

Best wishes,
MetabERN Patient Board steering committee POs and Dr Christina Lampe

1. Patient Organisation‘s name

2. Country

3. Covering which disease/s:

4. Have you been contacted by/do you know about metabolic patients, who are infected by COVID-19?

- ○ Yes
- ○ No

5. If yes,

how many adults/children?

how many sick/asymptomatic?

which metabolic disease are they suffering from?

6. Do you have any restrictions (issued e.g by health authorities/government) due to COVID-19 on a

	Yes/No
National level	
Regional level	

7. If yes, which restrictions (if no, please skip this question):

- ☐ not allowed to leave the house
- ☐ going out only with the family
- ☐ going out in small groups (more than 2 people)
- ☐ medical restrictions

Other (please specify)

8. Are you/patient members in close contact with your specialised centers/HCP about the COVID-19 crisis?

- ☐ Yes
- ☐ No
- ☐ I don't know

9. If yes:

- ○ Every day
- ○ Three to Four times per week
- ○ Twice a week
- ○ Once a week

Other (please specify)

10. In your country: how many hospital centers that are following your patient members are in contact with you in your capacity as a patient representative?

- ☐ All of them
- ☐ Most of them (more than 50%)
- ☐ A few (less than 50%)
- ☐ None

If none, do you think contact is being made directly with patients/caregivers?

11. Have any of these centers put in place measures or alternative care pathways to prevent COVID-19 infection in rare metabolic disease patients (e.g. teleconsultation)?

- ☐ Yes, all of them
- ☐ Yes, some of them
- ☐ Almost none
- ☐ None
- ☐ I don't know

If yes, please specify which

12. Are your members experiencing changes with the following:

	Follow up visits at your specialised center?	Follow up visits for clinical trials at your specialised center?
Answer		

13. If yes to either of the above, which changes (done by the center)

- ☐ Cancelling outpatient stays
- ☐ Cancelling inpatient stays
- ☐ Postponing appointments

14. Are you informed by your specialised centers about COVID-19 and its risk for your patients?

- ◯ Yes
- ◯ No

15. Have you shared this information with your members/patients?

- ◯ Yes
- ◯ No

16. If yes:

	Unhelpful	Somewhat helpful	Helpful	Very helpful
How helpful have your members found the information?	◯	◯	◯	◯

17. Was your association involved in the development of any informative materials?

- ◯ Yes
- ◯ No

If yes, are you willing to share it with us?

18. Are there any concrete recommendations issued by the medical community for your patient members/patient group?

- ◯ Yes
- ◯ No

19. If yes, what are the recommendations about:

- ☐ Risk of contracting the virus
- ☐ Hygiene/prophylaxis
- ☐ Work/Sick leave
- ☐ Planned follow up visits
- ☐ Clinical trials
- ☐ Treatment
- ☐ Therapies (physiotherapy, speech therapy, ERT)
- ☐ Vaccinations

Other (please specify)

20. Is there any change in treatment due to the COVID-19?

- ☐ Yes
- ☐ No

21. If yes, what are the changes:

- [ ] Discontinuation
- [ ] Prolonging the time between treatments
- [ ] Changing to home treatment
- [ ] Problems to get medication
- [ ] Continuing clinical trials
- [ ] The disease(s) we represent still do not have an approved therapy (in our country)

22. Are therapies such as physiotherapy, speech therapy, ERT, etc continued?

- [ ] Yes
- [ ] No
- [ ] I don't know

23. If yes,

- [ ] Postponed with a date
- [ ] Postponed without a date
- [ ] Cancelled

Other (please specify)

24. Is there any change in planned surgeries?

- [ ] Yes
- [ ] No
- [ ] I don't know

25. If yes:

- ○ Postponed with a date
- ○ Postponed without a date yet
- ○ Cancelled

Other (please specify)

26. Are your members/patients aware of what to do in emergencies (surgery, acute infections)?

- [ ] Yes
- [ ] No

Other (please specify)

27. Is there any psychological support for patients that are scared about COVID-19?

- [ ] Yes
- [ ] No

28. If yes, by whom?

- [ ] Physician
- [ ] Psychologist
- [ ] Social worker
- [ ] Your association

Other (please specify)

29. How?

- [ ] By Phone
- [ ] By Mail/Email
- [ ] By Video

Other (please specify)

30. How frequently are they available?

- ◯ 24 hours
- ◯ Only during open hours
- ◯ To make an appointment

31. How concerned is the patient community you represent at this point?

	Not worried at all	Somewhat worried	Neither worried nor unworried	Worried	Extremely worried
Having (or having the possibility) your therapy suspended	◯	◯	◯	◯	◯
Not having access to the same standard quality of care as before	◯	◯	◯	◯	◯
Anxiety and mental health issues related with the quarantine and current medical care limitations	◯	◯	◯	◯	◯
The possibility of getting COVID-19	◯	◯	◯	◯	◯
The additional strain being put on family caregivers	◯	◯	◯	◯	◯
Patients/caregivers not having access to reliable and comprehensible information	◯	◯	◯	◯	◯

32. Adult patients:

	Are they allowed to change to work at home due to the risk of getting infected?	Is there any problem to get a sick leave due to the increased risk?	Is there any changes in the daily care? (social services, day service)
Answer			

Other (please specify)
